# Positive mental health predicts amelioration of suicidal ideation in patients undergoing cognitive behavioral therapy

**DOI:** 10.3389/fpsyt.2025.1571300

**Published:** 2025-07-21

**Authors:** Tobias Teismann, Sören Friedrich, Jürgen Margraf

**Affiliations:** ^1^ Mental Health Research and Treatment Center, Ruhr-Universität Bochum, Bochum, Germany; ^2^ DZPG (German Center for Mental Health), partner site Bochum/Marburg, Bochum, Germany; ^3^ Department of Adolescent Psychology and Psychotherapy, Albert-Ludwigs-Universität Freiburg, Freiburg, Germany

**Keywords:** positive mental health, cognitive-behavioral therapy, suicidal ideation, suicide attempts, resilience

## Abstract

**Background:**

Positive mental health has been shown to confer resilience against suicidal ideation and behavior. Yet, studies on treatment seeking populations are rare.

The aim of the present study was to investigate, whether PMH predicts the amelioration of suicidal ideation in patients undergoing psychotherapy.

**Method:**

Data from *N* = 959 outpatients (61.2% female; age: *M(SD)* = 36.58 (13.29), range: 18–82 years), who took part in a pretreatment and a posttreatment assessment after 12 sessions of cognitive behavioral therapy, were included. Self-report measures of positive mental health, depression, and suicidal ideation/behavior were used.

**Results:**

Pretreatment PMH was shown to predict posttreatment suicidal ideation – after controlling for age, gender, pretreatment depression, suicidal ideation and lifetime suicide attempts.

**Discussion:**

Positive mental health might be understood as a protective factor in dealing with suicidal ideation. Findings underscore the need to focus on positive mental health in the risk-assessment and treatment of suicidal patients.

## Background

Positive mental health (PMH), that is, high levels of subjective and psychological well-being (1) has been shown to protect against suicidal ideation in a variety of studies (2): As such, it was shown that PMH - as assessed with the Positive Mental Health (PMH) Scale (3) - as well as social support predict the remission of suicidal ideation over the course of 17 months, whereas severity of psychopathology, life satisfaction and self-efficacy were not predictive of the course of suicide ideation (4). In a series of further studies PMH was found to moderate the relationship between different risk factors (e.g., depression, stressful life events) and suicidal ideation (2), as well as the relationship between suicidal ideation and suicide attempts (5). Corresponding effects were shown in Western and Eastern samples (2): In a study comparing the buffering qualities of PMH in German and Chinese students, only PMH, but neither self-efficacy, satisfaction with life, social support nor stress resistance moderated the effects of depression on suicide ideation in the samples from both countries (6). Of note, PMH and suicide ideation are not mutually exclusive, but exist simultaneously (7): Thus, PMH and suicidal ideation/behavior are not opposite poles of a single dimension, but form two independent factors of mental health/mental illness [cf. dual-factor models of mental health (1, 8)].

In sum, there are many studies highlighting the protective qualities of PMH, yet, compared to other protective factors (9), PMH has rather rarely been studied; even fewer studies have been conducted on treatment seeking populations and no study by now has investigated whether PMH predicts the amelioration of suicidal ideation in patients undergoing psychotherapy. Still, one study found that PMH predicts remission of suicidal ideation in an observational study (2) and another study found positive affectivity – a construct closely associated with PMH – to be associated with fewer suicidal symptoms following treatment (10). On this background, the present study aims to investigate whether PMH predicts posttreatment suicidal ideation in patients undergoing cognitive-behavioral therapy (CBT), while controlling for factors known to be associated with suicidal ideation/behavior within treatment [i.e. pretreatment depression, suicidal ideation and lifetime suicide attempts (11)].

## Materials and methods

### Participants

The study sample comprises *N*=959 outpatients (61.2% female; age: *M(SD)* = 36.58 (13.29), range: 18–82 years) undergoing cognitive-behavioral therapy (CBT) at an outpatient university clinic in the Ruhr-area in Germany between March 2017 and December 2023. Assessments were conducted prior to treatment (T1) and again at a post-treatment assessment after 12 therapy sessions (T2; mean time between T1 and T2: *M* = 5.60 month, *SD* = 2.54; number of therapy sessions: *M* = 11.87 sessions, *SD* = 0.77), which corresponds to a short-term therapy within the German insurance system. The most common primary diagnoses categories according to the International Classification of Diseases (ICD-10) were neurotic, stress and somatoform disorders (F4: 48%) as well as affective disorders (F3: 42.5%). All patients were Caucasian. Treatments were conducted by clinical psychologists in advanced CBT training, who provided treatment under close-meshed CBT supervision (every fourth session with a licensed CBT-supervisor). Treatments generally followed published CBT guidelines for each disorder, but were, in most cases, less standardized than in RCTs. Treatments were paid for by the German health insurance system.

All patients provided informed consent prior to participation. In order to assure a standard of quality, all clients seeking help at the clinic are required to fill out questionnaires prior to their intake. This study was reviewed and approved by the local Ethics Committee (318/2016).

### Measures

#### Suicide ideation and behavior scale [SIBS]

The SIBS (12) comprises six items assessing suicidal ideation, suicidal intent, suicidal impulses and suicide planning within the last 4 weeks. Items are rated on a 6-point Likert-type scale (0= *never*, 5=*many times a day*). Three further items assess the occurrence of a suicide attempt within the last four weeks as well as occurrence/frequency of lifetime suicide attempts. Excellent internal consistency has been demonstrated in German samples: *α*=.873 (12). Internal consistency was *α*=.812 (T1) and *α*=.863 (T2) in the current sample.

#### Positive mental health scale [PMHS]

The PMHS (3) assesses aspects of subjective and psychological well-being across nine items (e.g. “I feel that I am actually well equipped to deal with life and its difficulties”) rated on a scale ranging from 1 (*do not agree*) to 4 (*agree*), with higher scores indicating greater positive mental health. Unidimensional structure, good convergent and discriminant validity as well as high internal consistency (*α* >.81) have been demonstrated in various populations (3). Accordingly, Cronbach’s alpha was good in the current study: *α*=.90.

#### Depression-anxiety-stress scales – depression subscale [DASS-D]

The DASS-D (13) measures symptoms of depression over the past week with fourteen items (e.g. “I felt that life was meaningless”). All items are rated on a 4-point Likert-type scale (0 = *did not apply to me at all*, 3 = *applies to me very much or most of the time*). Higher sum scores indicate more severe symptoms. The subscale has been shown to have high internal consistency. Accordingly, internal consistency was good in the current sample: DASS-D: *α*= .95.

### Statistical analyses

Statistical analyses were conducted using SPSS 29. Changes in suicidal ideation from T1 to T2 were analyzed using a *t*-test for dependent samples and the effect size Cohens *d*. To identify significant predictors of suicidal ideation (SIBS) at the post-treatment assessment (T2), a 2-step hierarchical regression analyses was calculated with self-reported posttreatment suicidal ideation as dependent variable. Age, gender, DASS-D (T1), SIBS (T1), and lifetime suicide attempts (yes/no) were entered in step one and PMHS (T1) in the second step. There was no violation of the multicollinearity assumption as all values of tolerance were >.25, and all variance inflation factor values were <5.

## Results

In total, 42.2% patients (*n*=405) reported current suicidal ideation (SIBS≥1) at the pre-treatment assessment and 35.3% patients (*n*= 339) at the post-treatment assessment; 8.3% (*n*=80) reported at least one lifetime suicide attempt. A *t*-test for dependent samples revealed that suicidal ideation decreased from pre-treatment (*M* = 1.55, *SD* = 0.09) to post-treatment (*M* = 1.23, *SD* = 0.09), *t*(958) = 4,185, *p* <.001, *d* = .14.

**Table 1** shows the results of the hierarchical linear regression analyses with posttreatment suicidal ideation as criterion variable. Positive mental health predicted post-treatment suicidal ideation – after control of age, gender, pretreatment depression, suicidal ideation and lifetime suicide attempts. The result pattern remained the same, when the regression analysis was conducted only with data from those patients (*n*=405) who suffered from suicidal ideation at the pretreatment assessment.

**Table 1:** Prediction of posttreatment suicidal ideation (*n* = 959).

	Model 1				Model 2			
Pre-treatment characteristics	B	T	Beta	p	B	T	Beta	p
Age	-.004	-0.69	-.02	.491	-.005	-1.02	-.02	.308
Gender	-.088	-0.62	-.02	.531	-.098	-0.69	-.02	.488
DASS-D (T1)	.001	0.01	.01	.859	-.014	-1.47	-.05	.142
SIBS (T1)	.642	24.61	.67	.000	.638	24.49	.66	.000
Lifetime suicide attempts (yes/no)	.946	3.70	.09	.000	.924	3.62	.08	.000
PMHS (T1)	**-**	**-**		**-**	-.044	-2.49	-.08	.013
**Model**	**Adj. R^2^= .482** ***F*(5, 953)=179.21** ***p*<.001**				**Adj. R^2^ = .485** ***F*(6, 952)=151.20** ***p*<.001**			
**Change in R^2^**	**0.485** ***p*<.001**				**0.003** ***p*<.013**			

DASS-D, Depression Anxiety Stress Scales – Depression Subscale; PMHS, Positive Mental Health Scale; SIBS, Suicide Ideation and Behavior Scale.

In a set of exploratory analyses it was furthermore tested whether the result pattern stayed the same, when patients suffering either from unipolar depression (*n* = 399) or from an anxiety disorder (*n* = 205) where analyzed separately. Other diagnoses were too rare to carry out separate analyses. As can be seen in **Table 2** the result pattern generally remained the same when patients suffering from unipolar depression were analyzed. However, in patients suffering from an anxiety disorder pretreatment PMH was not predictive of posttreatment suicidal ideation (see **Table 3**).

**Table 2:** Prediction of posttreatment suicidal ideation in patients suffering from unipolar depression (*n* = 399).

	Model 1				Model 2			
Pre-treatment characteristics	B	T	Beta	p	B	T	Beta	p
Age	-.017	-2.09	-.07	.037	-.020	-2.50	-.02	.013
Gender	.131	0.58	-.02	.561	.094	0.42	.09	.675
DASS-D (T1)	-.018	-1.51	-.06	.131	-.051	-3.29	-.05	.001
SIBS (T1)	.691	18.20	.73	.001	.690	18.41	.69	.001
Lifetime suicide attempts (yes/no)	.527	1.40	.05	.162	.406	1.08	.41	.278
PMHS (T1)	**-**	**-**		**-**	-.096	-3.38	-.09	.001
**Model**	**Adj. R^2^= .520** ***F*(5, 393)=87.17** ***p*<.001**				**Adj. R^2^ = .532** ***F*(6, 392)=76.49** ***p*<.001**			
**Change in R^2^**	**0.520** ***p*<.001**				**0.013** ***p*<.001**			

DASS-D, Depression Anxiety Stress Scales – Depression Subscale; PMHS, Positive Mental Health Scale; SIBS, Suicide Ideation and Behavior Scale.

**Table 3:** Prediction of posttreatment suicidal ideation in patients suffering from anxiety disorders (*n* = 205).

	Model 1				Model 2			
Pre-treatment characteristics	B	T	Beta	p	B	T	Beta	p
Age	.009	0.80	.03	.420	.009	0.78	.03	.431
Gender	-.448	-1.60	-.07	.111	-.459	-1.63	-.07	.104
DASS-D (T1)	.002	0.11	.01	.912	-.008	-0.38	-.02	.698
SIBS (T1)	.789	13.23	.71	.001	.787	13.18	.70	.001
Lifetime suicide attempts (yes/no)	1.952	2.87	.14	.005	1.96	2.88	.14	.004
PMHS (T1)	**-**	**-**		**-**	-.024	-0.66	-.05	.507
**Model**	**Adj. R^2^= .571** ***F*(5, 199)=55.39** ***p*<.001**				**Adj. R^2^ = .570** ***F*(6, 198)=46.10** ***p*<.001**			
**Change in R^2^**	**0.571** ***p*<.001**				**0.001** ***p* =.507**			

DASS-D, Depression Anxiety Stress Scales – Depression Subscale; PMHS, Positive Mental Health Scale; SIBS, Suicide Ideation and Behavior Scale.

## Discussion

The present study found pretreatment positive mental health to predict posttreatment suicidal ideation in patients undergoing routine-care CBT. This result complements previous research which found PMH to confer resilience against suicidal ideation/behavior (2), to predict remission of suicidal ideation in an observational study (4) and to predict treatment outcome in patients receiving CBT more generally (14, 15). It must be highlighted that pretreatment PMH predicted posttreatment suicidal ideation over and above pretreatment suicidal ideation and lifetime suicide attempts – two well-established risk-factors for suicidal ideation and behavior within inpatient and outpatient treatments (cf. 11). This indicates that the PMH scale captures facets of positive mental health that are of particular importance for dealing with adversity.

One may speculate that positive mental health translates into more frequent everyday positive affect (16), which in turn has been shown to broaden individuals’ mindsets in ways that, help to build personal resources and to deal with lifes` negative challenges (17). In consequence, patients with heightened levels of PMH might be more attuned to treatment and therefore take more benefit from psychotherapy. It might also be that PMH is associated with a higher salience of reasons to live and thus contributes to a faster resolution of suicidal thoughts. And finally, PMH and/or positive emotions may counter suicidal cognitions by facilitating sufficient attentional disengagement from negative stimuli to allow distraction from and revaluation of stressful internal and external circumstances. The mechanisms by which PMH influences the treatment response should be investigated in more detail in future studies. In terms of clinical implications, the results of the current study add to previous findings suggesting that it could be important to account for the presence of PMH in addition to risk factors, when assessing individuals at risk for suicide. In the treatment of suicidal patients, a focus on resources and life-affirming aspects should be a matter of course. In addition, the Broad Minded Affective Coping (BMAC) technique, a brief positive mental imagery intervention (18), or other forms of positive psychology interventions (19) could be used to promote aspects of positive mental health in suicidal individuals. On a theoretical level, the current results underscore the necessity that theoretical models of suicide ideation/behavior should strive to integrate both pathogenetic and protective factors.

It should be noted however, that although a significant influence of positive mental health on suicidal ideation was found in the current study, this effect is only of small size. Therefore, it must be warned against overestimating the importance of positive mental health. Second, PMH was only shown to predict posttreatment suicidal ideation in patients suffering from unipolar depression, but not in patients suffering from anxiety disorders. It can be assumed that facets of positive mental health such as self-acceptance and self-efficacy (20) represent a stronger counterweight to depressive symptoms (such as hopelessness and despondency) than to anxiety symptoms (such as avoidance behavior) and that PMH therefore has a protective effect against suicidal ideation, especially in the context of unipolar depression. However, this pattern of findings needs to be replicated before far-reaching conclusions can be drawn. Third, as an outpatient sample was studied, levels of suicide ideation were rather modest. As such, it is unclear to what extent the results would generalize to high risk samples. Future studies should therefore include inpatient samples or patients in specialized crisis centers. Fourth, although PMH was found to predict reductions in suicidal ideation, the correlational nature of the study precludes firm conclusions about causality. Fifth, the study focuses only on pre/post-treatment outcomes but no long-term follow-up assessments. Finally, all patients in the current study received a CBT treatment. It therefore remains unclear, if findings would generalize to longer observation periods and/or to different treatment programs.

## Conclusion

Taken together, the current study underscores the importance of positive mental health as a protective or resilience factors against suicide ideation.

## Data Availability

The raw data supporting the conclusions of this article will be made available by the authors, without undue reservation.
